# The semisynthetic flavonoid monoHER sensitises human soft tissue sarcoma cells to doxorubicin-induced apoptosis via inhibition of nuclear factor-*κ*B

**DOI:** 10.1038/sj.bjc.6606065

**Published:** 2011-01-18

**Authors:** H Jacobs, A Bast, G J Peters, W J F van der Vijgh, G R M M Haenen

**Affiliations:** 1 Department of Pharmacology and Toxicology, Faculty of Health, Medicine and Life Sciences, NUTRIM School for Nutrition, Toxicology and Metabolism, Maastricht University Medical Centre+, P.O. Box 616, 6200 MD, Maastricht, The Netherlands; 2 Department of Medical Oncology, VU University Medical Center, De Boelelaan 1117, 1081 HV, Amsterdam, The Netherlands

**Keywords:** soft tissue sarcoma, doxorubicin, monoHER, glutathione, nuclear factor-*κ*B, apoptosis

## Abstract

**Background:**

Despite therapeutic advances, the prognosis of patients with metastatic soft tissue sarcoma (STS) remains extremely poor. The results of a recent clinical phase II study, evaluating the protective effects of the semisynthetic flavonoid 7-mono-O-(*β*-hydroxyethyl)-rutoside (monoHER) on doxorubicin-induced cardiotoxicity, suggest that monoHER enhances the antitumour activity of doxorubicin in STSs.

**Methods:**

To molecularly explain this unexpected finding, we investigated the effect of monoHER on the cytotoxicity of doxorubicin, and the potential involvement of glutathione (GSH) depletion and nuclear factor-*κ*B (NF-*κ*B) inactivation in the chemosensitising effect of monoHER.

**Results:**

MonoHER potentiated the antitumour activity of doxorubicin in the human liposarcoma cell line WLS-160. Moreover, the combination of monoHER with doxorubicin induced more apoptosis in WLS-160 cells compared with doxorubicin alone. MonoHER did not reduce intracellular GSH levels. On the other hand, monoHER pretreatment significantly reduced doxorubicin-induced NF-*κ*B activation.

**Conclusion:**

These results suggest that reduction of doxorubicin-induced NF-*κ*B activation by monoHER, which sensitises cancer cells to apoptosis, is involved in the chemosensitising effect of monoHER in human liposarcoma cells.

Soft tissue sarcomas (STSs) comprise a rare and diverse group of malignancies from mesenchymal origin, accounting for ∼1% of all adult malignancies (Jemal *et al*, 2009). The only single agents that are consistently associated with response rates of about 25% in metastatic STS are doxorubicin and ifosfamide (Santoro *et al*, 1995; Keohan and Taub, 1997).

In a clinical phase II study with metastatic cancer patients (Bruynzeel *et al*, 2007), evaluating the protective effects of the semisynthetic flavonoid 7-mono-O-(*β*-hydroxyethyl)-rutoside (monoHER) on doxorubicin-induced cardiotoxicity (Bast *et al*, 2007), we unexpectedly observed that of the four patients diagnosed with STS, three experienced objective remissions, whereas the fourth had stable disease. This observed 75% response rate is much better than the expected 25%. This prompted us to further study the sensitising effect of monoHER on doxorubicin-induced cytotoxicity.

MonoHER, like other flavonoids, may have an influence on the pathways that are involved in the development of resistance against doxorubicin (Kachadourian and Day, 2006; Sarkar and Li, 2007). Nuclear factor-*κ*B (NF-*κ*B) is one of the key factors involved in the development of chemoresistance (Karin, 2006; Sarkar and Li, 2008; Baud and Karin, 2009). It has been reported that chemotherapeutic agents, including doxorubicin, induce the activation of NF-*κ*B in cancer cells (Chuang *et al*, 2002), thereby inducing survival signals that inhibit apoptosis and promote cancer cell growth. Besides NF-*κ*B activation, increased glutathione (GSH) levels in cancer cells have also been associated with multidrug resistance of many tumours, as GSH can conjugate with the chemotherapeutic agent, leading to drug inactivation and excretion (Estrela *et al*, 2006).

Therefore, to support our clinical finding, we evaluated the effects of monoHER and doxorubicin on tumour cell growth, apoptosis, intracellular GSH levels and NF-*κ*B activation in human STS cell lines.

## Materials and methods

### Cell culture and reagents

The human STS cell lines SKUT-1 (leiomyosarcoma), SKLMS-1 (leiomyosarcoma), HT-1080 (fibrosarcoma) and WLS-160 (liposarcoma) (Medical Oncology, VU University medical center, Amsterdam, the Netherlands) were cultured under standard conditions.

Novartis Consumer Health (Nyon, Switzerland) kindly provided 7-mono-O-(*β*-hydroxyethyl)-rutoside (monoHER). From Sigma (St Louis, MO, USA), L-buthionine (SR)-sulphoximine (BSO) was purchased and doxorubicin HCl (2 mg ml^−1^) was obtained from Pharmacia Upjohn BV (Woerden, the Netherlands). All other chemicals were of analytical grade.

### Cell growth inhibition by sulphorhodamine B assay

The human STS cell lines were either untreated or pretreated with 50 *μ*M monoHER. After 1 h of incubation, medium was removed and replaced with fresh medium containing different concentrations of doxorubicin (0.001–10 *μ*M). After 72 h of incubation, cell viability was examined by the sulphorhodamine B assay (Vichai and Kirtikara, 2006).

### Caspase-3/7 assay for detecting apoptosis

WLS-160 cells were either untreated or pretreated with 50 *μ*M monoHER for 1 h, and then exposed to 10 *μ*M doxorubicin for an additional 6 h. After treatment, activation of caspase-3/7 was quantified by the Caspase-Glo 3/7 assay kit (Promega Corporation, Madison, WI, USA).

### Measurement of intracellular GSH

WLS-160 cells were incubated with 50 *μ*M monoHER or 50 *μ*M BSO for 1, 6 or 24 h. After incubation, cells were washed with PBS, harvested by treatment with trypsin-EDTA (Gibco, Paisley, UK) and washed again with ice-cold PBS (Gibco). After centrifugation, cells were resuspended in ice-cold extraction buffer (0.1% Triton X-100 and 0.6% SSA, Sigma) and sonicated in icy water for 2–3 min. The extracts were used for determination of intracellular GSH using an enzymatic recycle method (Rahman *et al*, 2006).

### Nuclear factor-*κ*B measurement

WLS-160 cells were treated with 10 *μ*M doxorubicin for 1.5, 3, 6 or 24 h. In another experiment, the cells were either untreated or pretreated for 1 h with 50 *μ*M monoHER before doxorubicin exposure (10 *μ*M; 6 h). Nuclear factor-*κ*B expression was determined in nuclear extracts of the cells (Hofmann *et al*, 1999) using the TransAM NF-*κ*B p50 transcription factor assay kit (Active Motif, Rixensart, Belgium). Protein concentrations were determined using the method of Bradford (Biorad, Veenendaal, the Netherlands).

### Statistical analysis

All experiments were performed, at least, in triplicate. Results are given as mean±s.d. or as a typical example. The statistical significance of the differences between experimental groups and controls was determined by Student's *t*-test. *P*-values ⩽0.05 were considered statistically significant.

## Results

**Figure 1:**
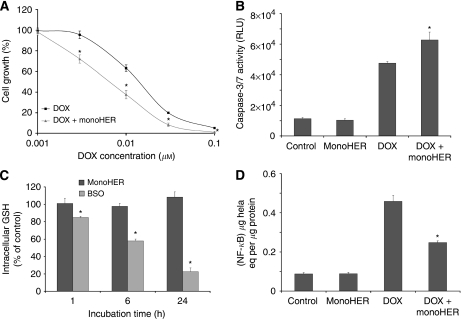
Effect of the semi-synthetic flavonoid monoHER on the cytotoxic effects of doxorubicin in the liposarcoma cell line WLS-160. (**A**) MonoHER pretreatment (50 *μ*M; 1 h) significantly enhances the cell growth inhibition induced by doxorubicin (0.001–0.1 *μ*M; 72 h) (mean±s.d. ^*^P⩽0.05 compared with doxorubicin treatment). (**B**) MonoHER pretreatment (50 *μ*M; 1 h) significantly enhances the apoptosis induced by doxorubicin (10 *μ*M; 6 h) (mean±s.d. ^*^P⩽0.05 compared with doxorubicin treatment). Relative light units (RLUs); doxorubicin (DOX). (**C**) MonoHER treatment (50 *μ*M; 1, 6 and 24 h) has no effect on the intracellular GSH concentration, whereas BSO (50 *μ*M; 1, 6 and 24 h) reduces GSH levels in a time-dependent manner (mean±s.d. ^*^P⩽0.05 compared with control). (**D**) MonoHER pretreatment (50 *μ*M; 1 h) significantly prevents doxorubicin-induced (10 *μ*M; 6 h) NF-*κ*B activation (mean±s.d. ^*^P⩽0.05 compared with doxorubicin treatment).

As shown in Table 1, monoHER pretreatment did not significantly influence the antitumour activity of doxorubicin in SKUT-1, SKLMS-1 and HT-1080 cells. However, pretreatment of the liposarcoma cell line, WLS-160, with 50 *μ*M monoHER for 1 h before doxorubicin exposure shifted the growth inhibition curve of doxorubicin to the left (Figure 1A). MonoHER alone did not affect tumour growth. These results indicate that monoHER potentiated the antitumour activity of doxorubicin in WLS-160 cells.

The effect of monoHER on doxorubicin-induced apoptosis in WLS-160 cells as assessed by caspase-3/7 activation is shown in Figure 1B. Doxorubicin treatment (10 *μ*M; 6 h) strongly induced caspase-3/7 activity. Pretreatment of the cells with 50 *μ*M monoHER for 1 h before doxorubicin exposure significantly enhanced the doxorubicin-induced caspase-3/7 activation. MonoHER alone had no effect on caspase-3/7 activity. These findings indicate that monoHER sensitised these cancer cells to doxorubicin-induced apoptosis.

As shown in Figure 1C, treatment of WLS-160 cells with 50 *μ*M monoHER for 1, 6 or 24 h did not affect intracellular GSH levels. In contrast, the GSH synthesis inhibitor BSO induced a time-dependent GSH depletion in WLS-160 cells. Moreover, BSO also enhanced the antitumour activity of doxorubicin (data not shown). These results indicate that intracellular GSH depletion can sensitise WLS-160 cells to doxorubicin-induced apoptosis. However, this mechanism is not involved in the chemosensitising effects of monoHER.

Doxorubicin treatment (10 *μ*M; 1.5, 3, 6 and 24 h) significantly induced NF-*κ*B DNA-binding activity in WLS-160 cells compared with untreated controls. Within the measured time range, NF-*κ*B activation was maximal at 6 h of drug exposure (data not shown). Figure 1D shows that pretreatment of cells with 50 *μ*M monoHER for 1 h before 6 h of doxorubicin exposure significantly reduced doxorubicin-induced activation of NF-*κ*B. MonoHER alone had no effect on the basal NF-*κ*B level in WLS-160 cells. These results suggest that reduction of doxorubicin-induced NF-*κ*B activation might be involved in the sensitising effect of monoHER on the antitumour effect of doxorubicin.

## Discussion

In this paper, we show that monoHER can enhance the cytotoxicity of doxorubicin, possibly via modulation of NF-*κ*B.

Four different human STS cell lines were investigated. Only in the liposarcoma cell line, WLS-160, pretreatment with monoHER resulted in a significantly greater inhibition of cancer cell growth. This different response on monoHER pretreatment between the different cell lines is possibly because of the known large diversity in STS and the subsequent response to chemotherapy (Van Glabbeke *et al*, 1999; Verweij, 2009). The molecular mechanism behind the observed sensitising effect of monoHER in the responding liposarcoma cell line, WLS-160, was further explored.

First, we investigated how cytotoxicity of doxorubicin and the chemosensitising effect of monoHER were mediated. Doxorubicin as a single agent activated caspase-3/7 activity and thus induced apoptosis in WLS-160 cells. Pretreatment of these cells with monoHER resulted in a significantly greater induction of apoptosis. These results suggest that the greater degree of cancer cell death by the combination of monoHER with doxorubicin is mediated by the induction of an apoptotic pathway.

As multidrug resistance of many cancer cells is associated with increased intracellular GSH levels, GSH depletion is a potential strategy to sensitise tumour cells to chemotherapeutics and modify drug resistance (Meister, 1991; Estrela *et al*, 2006). This can be achieved by inhibitors of GSH synthesis such as BSO, a selective and irreversible inhibitor of *γ*-glutamylcysteine synthase, the rate limiting enzyme of GSH synthesis (Han and Park, 2009). As also shown by our results, BSO efficiently enhances the effect of several chemotherapeutic drugs including doxorubicin (Vanhoefer *et al*, 1996). In addition, some flavonoids can also deplete GSH levels in cancer cells, thereby sensitising these cancer cells to chemotherapy (Kachadourian and Day, 2006; Kachadourian *et al*, 2007; Ramos and Aller, 2008). Because monoHER is able to form a GSH–monoHER adduct that might deplete cells from GSH (Jacobs *et al*, 2009), we examined whether monoHER can deplete GSH in WLS-160 cells. However, in contrast to BSO, monoHER did not significantly change GSH levels in WLS-160 cells, suggesting that the growth-inhibitory effect of monoHER is not mediated via GSH depletion.

**Figure 2:**
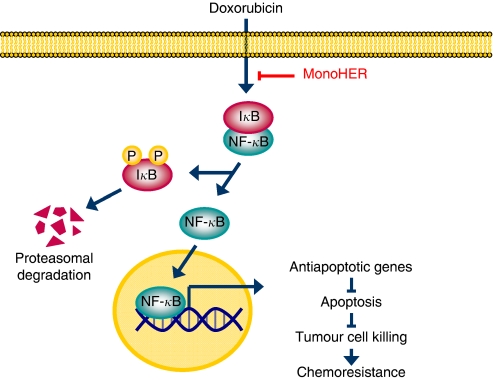
Suggested pathway illustrating the influence of monoHER on doxorubicin cytotoxicity in WLS-160 cells. Under resting conditions, NF-*κ*B is maintained in an inactive state in the cytoplasm via interaction with the inhibitory protein, I*κ*B. Doxorubicin can activate the NF-*κ*B pathway, which involves the phosphorylation, ubiquitination and proteasomal degradation of I*κ*B. Nuclear factor-*κ*B is then free to translocate to the nucleus where it facilitates the transcription of, for example, antiapoptotic genes, resulting in less tumour cell killing and the development of drug resistance. MonoHER is able to reduce this doxorubicin-induced NF-*κ*B activation, thereby sensitising WLS-160 cells to doxorubicin-induced apoptosis.

Another crucial factor involved in drug resistance is NF-*κ*B, an inducible and ubiquitously expressed transcription factor that regulates cell survival, inflammation and differentiation (Karin, 2006). Many chemotherapeutic agents induce the activity of NF-*κ*B, which causes drug resistance in cancer cells (Sarkar and Li, 2008). Because doxorubicin rapidly induced NF-*κ*B activity in WLS-160 cells within 6 h, inhibition of NF-*κ*B was expected to enhance the antitumour activity of doxorubicin, similar to several *in vitro* and preclinical *in vivo* studies in which the regulation of NF-*κ*B enhanced the efficacy of chemotherapy (Bava *et al*, 2005; Li *et al*, 2005; Nakanishi and Toi, 2005; Sung *et al*, 2007). Our results show that monoHER prevented the NF-*κ*B induction by doxorubicin in WLS-160 cells, suggesting that downregulation of NF-*κ*B activation by monoHER may be responsible for the sensitisation of these cancer cells to doxorubicin. Figure 2 further illustrates this mechanism.

In conclusion, monoHER enhanced the cytotoxicity of doxorubicin in the human liposarcoma cell line WLS-160. This potentiation was not mediated by GSH depletion, but monoHER reduced doxorubicin-induced NF-*κ*B activation, thereby sensitising tumour cells to apoptosis. Thus, the high response rate in the clinical phase II study may be mediated by reduction of doxorubicin-induced NF-*κ*B activation. For certain STS patients, monoHER might improve chemotherapy and even decrease systemic toxicity. Moreover, monoHER might also be valuable for the treatment of other tumours that have developed chemoresistance through NF-*κ*B activation. However, future studies are needed to further elucidate the value of adding monoHER to the chemotherapeutic treatment with doxorubicin.

## Figures and Tables

**Table 1:** IC_50_ values (*μ*M) of growth inhibition by doxorubicin^a^

**Human STS cell line**	**Doxorubicin**	**Doxorubicin + monoHER**
SKLMS-1	0.063±0.009	0.066±0.007
SKUT-1	0.048±0.008	0.045±0.009
HT-1080	0.024±0.005	0.027±0.006
WLS-160	0.016±0.003	0.0075±0.001^*^

Abbreviations: IC_50_=inhibitory concentration 50% monoHER**=**7-mono-O-(*β*-hydroxyethyl)-rutoside,

a Data are expressed as mean±s.d. ^*^P⩽0.05.

## References

[bib1] Bast A, Haenen GR, Bruynzeel AM, Van der Vijgh WJ (2007) Protection by flavonoids against anthracycline cardiotoxicity: from chemistry to clinical trials. Cardiovasc Toxicol 7: 154–159 17652822 10.1007/s12012-007-0018-0

[bib2] Baud V, Karin M (2009) Is NF-kappaB a good target for cancer therapy? Hopes and pitfalls. Nat Rev Drug Discov 8: 33–40 19116625 10.1038/nrd2781 PMC2729321

[bib3] Bava SV, Puliappadamba VT, Deepti A, Nair A, Karunagaran D, Anto RJ (2005) Sensitization of taxol-induced apoptosis by curcumin involves down-regulation of nuclear factor-kappaB and the serine/threonine kinase Akt and is independent of tubulin polymerization. J Biol Chem 280: 6301–6308 15590651 10.1074/jbc.M410647200

[bib4] Bruynzeel AM, Niessen HW, Bronzwaer JG, van der Hoeven JJ, Berkhof J, Bast A, van der Vijgh WJ, van Groeningen CJ (2007) The effect of monohydroxyethylrutoside on doxorubicin-induced cardiotoxicity in patients treated for metastatic cancer in a phase II study. Br J Cancer 97: 1084–1089 17940501 10.1038/sj.bjc.6603994 PMC2360436

[bib5] Chuang SE, Yeh PY, Lu YS, Lai GM, Liao CM, Gao M, Cheng AL (2002) Basal levels and patterns of anticancer drug-induced activation of nuclear factor-kappaB (NF-kappaB), and its attenuation by tamoxifen, dexamethasone, and curcumin in carcinoma cells. Biochem Pharmacol 63: 1709–1716 12007574 10.1016/s0006-2952(02)00931-0

[bib6] Estrela JM, Ortega A, Obrador E (2006) Glutathione in cancer biology and therapy. Crit Rev Clin Lab Sci 43: 143–181 16517421 10.1080/10408360500523878

[bib7] Han YH, Park WH (2009) The effects of N-acetyl cysteine, buthionine sulfoximine, diethyldithiocarbamate or 3-amino-1,2,4-triazole on antimycin A-treated Calu-6 lung cells in relation to cell growth, reactive oxygen species and glutathione. Oncol Rep 22: 385–391 19578781

[bib8] Hofmann MA, Schiekofer S, Isermann B, Kanitz M, Henkels M, Joswig M, Treusch A, Morcos M, Weiss T, Borcea V, Abdel Khalek AK, Amiral J, Tritschler H, Ritz E, Wahl P, Ziegler R, Bierhaus A, Nawroth PP (1999) Peripheral blood mononuclear cells isolated from patients with diabetic nephropathy show increased activation of the oxidative-stress sensitive transcription factor NF-kappaB. Diabetologia 42: 222–232 10064103 10.1007/s001250051142

[bib9] Jacobs H, van der Vijgh WJ, Koek GH, Draaisma GJ, Moalin M, van Strijdonck GP, Bast A, Haenen GR (2009) Characterization of the glutathione conjugate of the semisynthetic flavonoid monoHER. Free Radic Biol Med 46: 1567–1573 19272444 10.1016/j.freeradbiomed.2009.02.031

[bib10] Jemal A, Siegel R, Ward E, Hao Y, Xu J, Thun MJ (2009) Cancer statistics, 2009. CA Cancer J Clin 59: 225–249 19474385 10.3322/caac.20006

[bib11] Kachadourian R, Day BJ (2006) Flavonoid-induced glutathione depletion: potential implications for cancer treatment. Free Radic Biol Med 41: 65–76 16781454 10.1016/j.freeradbiomed.2006.03.002 PMC3983951

[bib12] Kachadourian R, Leitner HM, Day BJ (2007) Selected flavonoids potentiate the toxicity of cisplatin in human lung adenocarcinoma cells: a role for glutathione depletion. Int J Oncol 31: 161–168 17549417 PMC3983955

[bib13] Karin M (2006) Nuclear factor-kappaB in cancer development and progression. Nature 441: 431–436 16724054 10.1038/nature04870

[bib14] Keohan ML, Taub RN (1997) Chemotherapy for advanced sarcoma: therapeutic decisions and modalities. Semin Oncol 24: 572–579 9344324

[bib15] Li Y, Ahmed F, Ali S, Philip PA, Kucuk O, Sarkar FH (2005) Inactivation of nuclear factor kappaB by soy isoflavone genistein contributes to increased apoptosis induced by chemotherapeutic agents in human cancer cells. Cancer Res 65: 6934–6942 16061678 10.1158/0008-5472.CAN-04-4604

[bib16] Meister A (1991) Glutathione deficiency produced by inhibition of its synthesis, and its reversal; applications in research and therapy. Pharmacol Ther 51: 155–194 1784629 10.1016/0163-7258(91)90076-x

[bib17] Nakanishi C, Toi M (2005) Nuclear factor-kappaB inhibitors as sensitizers to anticancer drugs. Nat Rev Cancer 5: 297–309 15803156 10.1038/nrc1588

[bib18] Rahman I, Kode A, Biswas SK (2006) Assay for quantitative determination of glutathione and glutathione disulfide levels using enzymatic recycling method. Nat Protoc 1: 3159–3165 17406579 10.1038/nprot.2006.378

[bib19] Ramos AM, Aller P (2008) Quercetin decreases intracellular GSH content and potentiates the apoptotic action of the antileukemic drug arsenic trioxide in human leukemia cell lines. Biochem Pharmacol 75: 1912–1923 18359480 10.1016/j.bcp.2008.02.007

[bib20] Santoro A, Tursz T, Mouridsen H, Verweij J, Steward W, Somers R, Buesa J, Casali P, Spooner D, Rankin E (1995) Doxorubicin versus CYVADIC versus doxorubicin plus ifosfamide in first-line treatment of advanced soft tissue sarcomas: a randomized study of the European Organization for Research and Treatment of Cancer Soft Tissue and Bone Sarcoma Group. J Clin Oncol 13: 1537–1545 7602342 10.1200/JCO.1995.13.7.1537

[bib21] Sarkar FH, Li Y (2008) NF-kappaB: a potential target for cancer chemoprevention and therapy. Front Biosci 13: 2950–2959 17981768 10.2741/2900

[bib22] Sarkar FH, Li YW (2007) Targeting multiple signal pathways by chemopreventive agents for cancer prevention and therapy. Acta Pharmacol Sin 28: 1305–1315 17723164 10.1111/j.1745-7254.2007.00689.x

[bib23] Sung B, Pandey MK, Aggarwal BB (2007) Fisetin, an inhibitor of cyclin-dependent kinase 6, down-regulates nuclear factor-kappaB-regulated cell proliferation, antiapoptotic and metastatic gene products through the suppression of TAK-1 and receptor-interacting protein-regulated IkappaBalpha kinase activation. Mol Pharmacol 71: 1703–1714 17387141 10.1124/mol.107.034512

[bib24] Van Glabbeke M, van Oosterom AT, Oosterhuis JW, Mouridsen H, Crowther D, Somers R, Verweij J, Santoro A, Buesa J, Tursz T (1999) Prognostic factors for the outcome of chemotherapy in advanced soft tissue sarcoma: an analysis of 2185 patients treated with anthracycline-containing first-line regimens—a European Organization for Research and Treatment of Cancer Soft Tissue and Bone Sarcoma Group Study. J Clin Oncol 17: 150–157 10458228 10.1200/JCO.1999.17.1.150

[bib25] Vanhoefer U, Cao S, Minderman H, Toth K, Skenderis II BS, Slovak ML, Rustum YM (1996) d,l-Buthionine-(S,R)-sulfoximine potentiates *in vivo* the therapeutic efficacy of doxorubicin against multidrug resistance protein-expressing tumors. Clin Cancer Res 2: 1961–1968 9816155

[bib26] Verweij J (2009) Soft tissue sarcoma trials: one size no longer fits all. J Clin Oncol 27: 3085–3087 19451424 10.1200/JCO.2009.21.8180

[bib27] Vichai V, Kirtikara K (2006) Sulforhodamine B colorimetric assay for cytotoxicity screening. Nat Protoc 1: 1112–1116 17406391 10.1038/nprot.2006.179

